# Identification and Treatment of Intravascular Hypervolemia and Polycythemia in Patients With Heart Failure: Role of Therapeutic Phlebotomy

**DOI:** 10.1016/j.jaccas.2024.103077

**Published:** 2025-02-12

**Authors:** Mousab Al Abbas, Smitha Sagaram, Marc A. Silver

**Affiliations:** a Advanced Heart Failure Program, Banner University Medical Center, Phoenix, Arizona, USA; b Department of Medicine, University of Arizona, Phoenix, Arizona, USA; c Banner University Medical Center, Phoenix, Arizona, USA

**Keywords:** blood volume analysis, heart failure, phlebotomy, polycythemia

## Abstract

Intravascular hypervolemia with polycythemia is a blood volume phenotype in patients with heart failure that portends additional risks including thrombotic events. This paper presents 4 patients with heart failure with this blood volume phenotype and focuses on its identification and the role of therapeutic phlebotomy including patient selection and outcomes.

## Graphical Abstract

**Figure:**
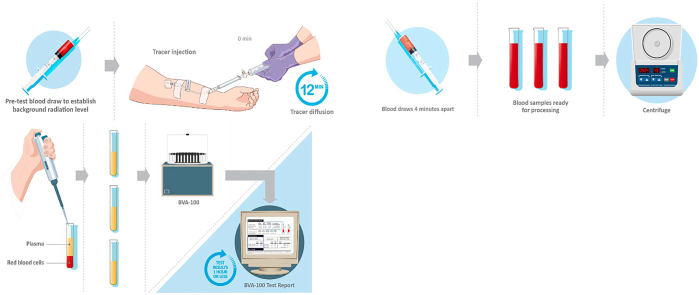

## Background

Total body fluid resides in 3 volume compartments: intracellular, interstitial, and intravascular. The intravascular volume compartment is tightly regulated and its constituent volumes (total blood volume, red blood cell volume, and plasma volumes) are within 8% to 10% variation of normal in health; dysregulation of these volumes is common in heart failure and contributes to the initiation and progression of heart failure syndrome.

**Take-Home Messages**

• Increased plasma volume may mask the polycythemia, and diuresis alone may lead to excess hemoconcentration.
• BVA evaluation is an extremely useful and specific diagnostic test to be considered in these patients.

**Figure 1:**
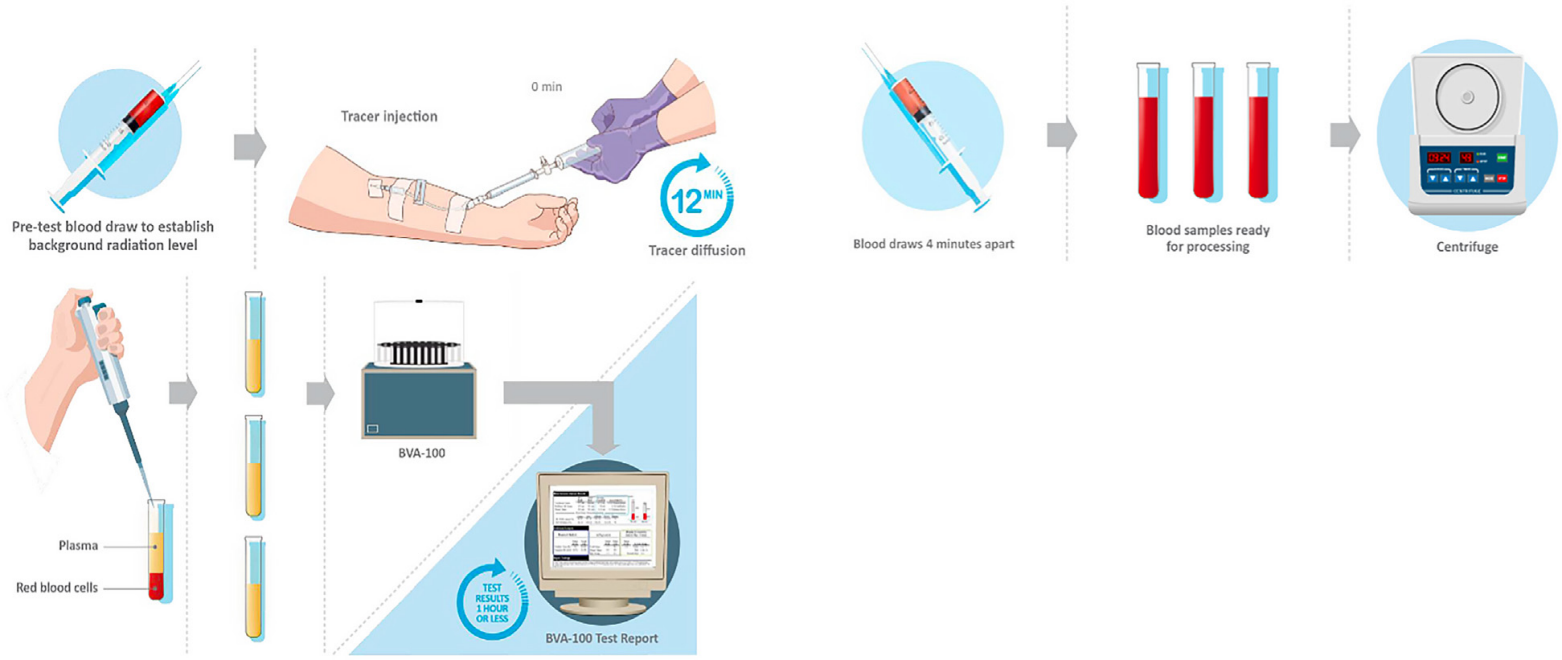
Methodology of Performing Blood Volume Analysis Total blood volume quantification analyses using the indicator-dilution principle using a standardized clinically available computer-based technique to administer intravenously microcurie dose iodinated (I^-131^) labeled albumin. Graphic courtesy of Daxor Corp.

Precise measurement of the intravascular volumes is done using an established single isotope radiolabeling technique known as blood volume analysis (BVA) (Figure 1). Using this technique, derangements of each of the volumes yield a patient-specific BVA phenotype which allows guided therapy.

Significant increases in total blood volume with an associated increase in red blood cell volume represents a hypervolemic/polycythemic phenotype. The BVA test also identifies a patient’s increased risk for thromboembolic events by also providing a normalized hematocrit (Hct) which is a calculated value of what the Hct would be if the total blood volume was corrected by removing only plasma (ie, diuresis), resulting in hemoconcentration of the remaining red blood cell volume.

## Objectives

Polycythemia with hypervolemia is a frequent intravascular blood volume phenotype in patients with heart failure.

BVA offers precise, otherwise undetected derangements and insights into the risk of thrombotic events.

Low volume phlebotomy (1-2 U) may improve symptoms and quality of life of patients with heart failure with significant polycythemia detected with BVA analysis.

## Methods

**Figure 2:**
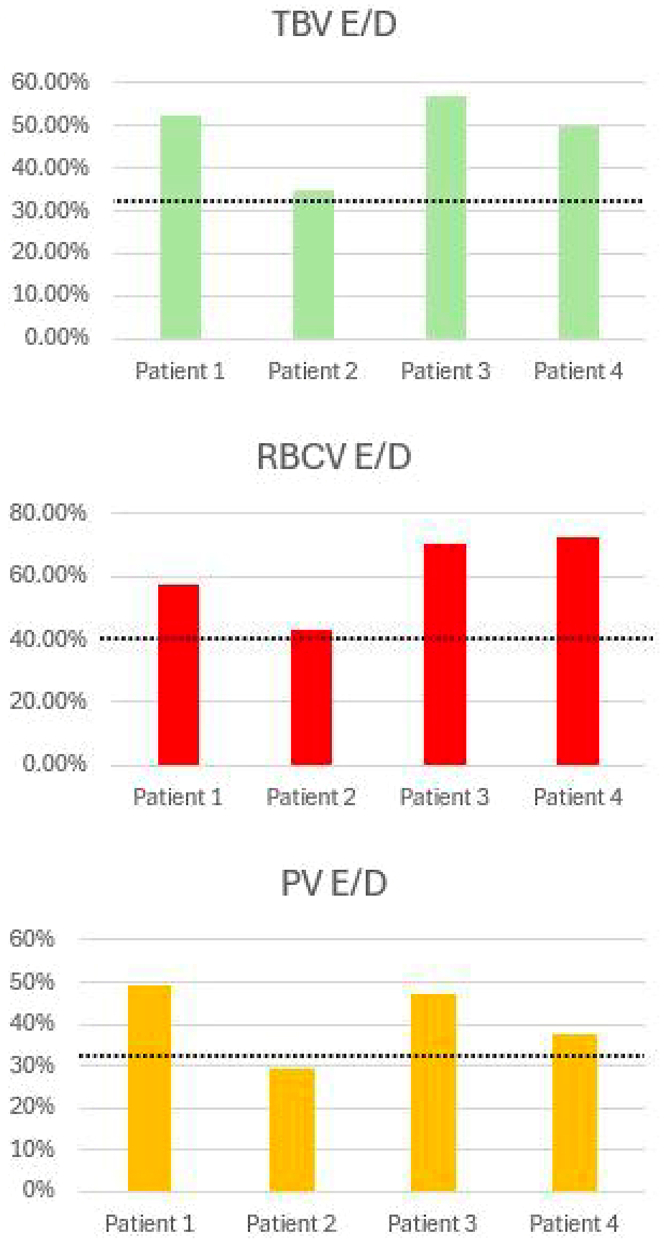
Intravascular Blood Volumes of 4 Patients Expressed as Excesses of Predicted Normal The excess of total blood volume (TBV), red blood cell volume (RBCV), and plasma volume (PV) of all patients is shown. The dashed lines represent the threshold of extreme excess. E/D = excess/deficit.

Of 179 advanced patients with heart failure undergoing BVA analysis (Daxor), 36 had hypervolemia/polycythemia phenotype (20%). Of these 36, 4 patients underwent 1 or more therapeutic phlebotomies based on functional status and risk related to their significantly elevated normalized Hct being >65%. Demographics and baseline clinical characteristics are shown in Table 1, and baseline BVA results are summarized in Table 2 and Figure 2.

**Table 1:** Summary of Demographics and Baseline Clinical Characteristics of Hypervolemic/Polycythemic Patients With Heart Failure Treated With Low Volume Therapeutic Phlebotomy

	Patient 1	Patient 2	Patient 3	Patient 4
Age, y	78	55	51	38
Sex	Male	Male	Male	Female
Weight, kg	119	127	104	117
Height, cm	175	190	180	165
BMI, kg/m^2^	38.9	35.2	32	43
Smoking history	Never	Never	Former smoker	Never
Illicit drug abuse	Never	Never	Never	Never
OSA	No	Yes	No	Yes
COPD	No	No	No	No
Testosterone use	Yes, gel supplements	No	Yes, 300 mg SQ weekly	No
JAK2 mutation	Negative	Negative	Negative	Negative
Etiology of HF	Severe AS and ischemic cardiomyopathy	Ischemic cardiomyopathy	Nonischemic cardiomyopathy	Peripartum cardiomyopathy
EF, %	50	25	40	33
NYHA functional class	III	IV	II	III
Presentation	Increasing exertional dyspnea, lower extremity edema, and orthopnea	Exertional dyspnea, PND, and orthopnea	Reported mild symptoms with full functional capacity	Chronic fatigue, dyspnea on exertion, and orthopnea
GDMT and diuretics	Carvedilol 25 mg twice a day Sacubitril/valsartan 24/26 mg every day Spironolactone 25 mg every day Empagliflozin 5 mg every day Torsemide 20 mg twice a day Isosorbide mononitrate 20 mg twice a day	Carvedilol 25 mg every day Sacubitril/valsartan 24/26 mg every day Empagliflozin 10 mg every day Spironolactone 25 mg every day Furosemide 20 mg twice a day	Carvedilol 25 mg twice a day Lisinopril 10 mg every day Spironolactone 25 mg every day	Metoprolol extended release 25 mg every day Losartan 25 mg every day Spironolactone 25 mg every day Empagliflozin 10 mg every day Bumetanide 1 mg 3 times a day
Antiplatelets and anticoagulants	Apixaban 5 mg twice a day	Clopidogrel 75 mg every day Apixaban 5 mg every day	___	___
Number of phlebotomy sessions	3	2	3	2

AS = aortic stenosis; BMI = body mass index; COPD = chronic obstructive pulmonary disease; EF = ejection fraction; GDMT = guideline-directed medical therapy; HF = heart failure; JAK-2 = Janus Kinase 2; OSA = obstructive sleep apnea; PND = paroxysmal nocturnal dyspnea; SQ = subcutaneous.

**Table 2:** Summary of the Patients’ Volume Status Measured by the BVA-100 Test (Baseline)

	Patient 1	Patient 2	Patient 3	Patient 4
TBV, mL	9219	9234	9007	6257
TBV E/D, %	52.4	34.9	56.8	50
RBCV, mL	3862	3972	3976	2705
RBCV E/D, %	57.5	43.2	70.6	72.5
PV, mL	5357	5262	5031	3822
PV E/D, %	49	29.3	47.3	37.4
Peripheral hematocrit, %	46.5	47.8	49	46
Normalized hematocrit, %	70.9	65.4	76.8	69
BVA-TBV	Hypervolemic	Hypervolemic	Hypervolemic	Hypervolemic
BVA-RBCV	Polycythemic	Polycythemic	Polycythemic	Polycythemic

	Blood Volume Interpretation Guideline				
	Normal	Mild	Moderate	Severe	Extreme
TBV, PV deviation (±), %	0-8	>8-16	>16-24	>24-32	>32
RBCV deviation (±), %	0-10	>10-20	>20-30	>30-40	>40

BVA = blood volume analysis; E/D = excess/deficit; PV = plasma volume; RBCV = red blood cell volume; TBV = total blood volume.

## Case 1

A 78-year-old man with a history of ischemic cardiomyopathy, heart failure with preserved ejection fraction, and prior aortic valve replacement due to aortic stenosis presented with increasing dyspnea on exertion and NYHA functional class III, with early satiety, +3 bilateral lower extremity edema, and orthopnea. Medication history included testosterone gel as a supplement. A BVA was ordered due to the patient’s symptoms and his testosterone use. The BVA showed significant hypervolemia/polycythemia along with a significant increase in normalized Hct (Table 2). The patient was evaluated by a hematology consultant who thought he had a secondary form of polycythemia related to exogenous testosterone use. We performed therapeutic phlebotomy; he underwent 3 supervised sessions of low-dose therapeutic phlebotomies (250-500 mL) over a 3-month period. After this management, the patient reported better exercise tolerance and functional capacity (NYHA functional class II) with significant improvement in his clinical volume status and quality of sleep.

## Case 2

A 55-year-old man presented to the advanced heart failure clinic with a history of ischemic cardiomyopathy, NYHA functional class IV, heart failure with reduced ejection fraction, prior pulmonary embolism, deep venous thrombosis, morbid obesity, and obstructive sleep apnea (OSA). He reported poor compliance with his nocturnal continuous positive airway pressure. He was complaining of dyspnea on exertion, orthopnea, and intermittent paroxysmal nocturnal dyspnea. Due to his symptoms, thrombosis history, and OSA, a BVA was performed which showed hypervolemia/polycythemia (Table 2).

We performed 2 supervised phlebotomies (250-500 mL) over a 4-month period while keeping the patient on his guideline-directed medical therapy (Table 1). After the initial phlebotomy, the patient reported significant improvement in his functional status, reported significant improvement in quality of sleep, and denied any dyspnea on exertion. He is currently NYHA functional class I.

## Case 3

A 51-year-old man presented to the heart failure clinic with a history of heart failure with reduced ejection fraction and obesity, pulmonary embolus, and deep venous thrombosis. The patient was mildly symptomatic with NYHA functional class II, yet active and denied severe dyspnea, orthopnea, or paroxysmal nocturnal dyspnea. He was taking subcutaneous testosterone supplements weekly. Despite mild symptoms, based on his history of testosterone use, the patient underwent BVA. It demonstrated hypervolemic/polycythemic phenotype, with extreme increase in his normalized Hct (Table 2). No medication changes were made. On recommendation from hematology and based on a persistent elevation of his peripheral Hct, he underwent 3 additional (250-500 mL) therapeutic phlebotomies, and guided by repeat BVA, normalized Hct declined to 51.7%. The patient continues working actively with good sleep and without any heart failure symptomatology with NYHA functional class I.

## Case 4

A 38-year-old woman with a history of peripartum cardiomyopathy, eclampsia, morbid obesity, deep vein thrombosis, and OSA presented to the advanced heart failure clinic complaining of dyspnea on exertion with NYHA functional class III, chronic fatigue, and orthopnea. Continuous positive airway pressure was not tolerated by the patient. A BVA revealed a hypervolemic/polycythemic phenotype with an associated elevated normalized Hct (Table 2). No changes in medications were made and the patient underwent 2 therapeutic phlebotomies. After the procedure, the patient reported significant improvement in her functional status (NYHA functional class I) and better sleep.

All 4 patients tolerated phlebotomy well, improved clinically, and had no hospitalization at 6 months and no thromboembolic events at 6 and 12 months.

## Discussion

The prevalence of polycythemia in the heart failure population (all phenotypes) with or without hypervolemia is estimated to be 10% to 12%.^1^ Additionally, polycythemia may be masked in hypervolemic patients with heart failure because the expansion of plasma volume may confound the measurements of Hct and hemoglobin^2^; therefore, the true Hct level is underestimated by the large plasma volume.

Primary polycythemia is usually caused by a myeloproliferative disorder, polycythemia vera, whereas secondary polycythemia is more common and can be caused by diuretic use, hypoxia, high altitude living, chronic obstructive pulmonary disease, smoking, or drugs (eg, testosterone, anabolic steroids).^3^

The patients were selected for consideration for phlebotomy based on their markedly increased normalized Hct driven by secondary causes.^3^ Cases 1 and 3 use testosterone supplements, whereas cases 2 and 4 had morbid obesity and OSA; however, all of them had obesity with an average BMI of 37.2 kg/m^2^. However, normalizing the volume without addressing the severely elevated normalized Hct may increase the risk of thrombosis in patients with an established thrombotic history.

In the ELITE II clinical trial, hemoglobin level independently predicted mortality in the heart failure population; either anemia or polycythemia resulted in higher mortality rates than patients with heart failure with normal hemoglobin levels.^4^

In polycythemia vera patients, keeping the Hct level below a target of 45% was associated with significantly lower incidence of cardiovascular and major thrombosis events than an Hct level of 45% to 50%.^5^ Additionally, secondary polycythemia in patients receiving testosterone treatment independently increases the risk of major adverse cardiovascular events and venous thromboembolism in the first year of therapy.^6^

## Conclusions

Hypervolemia/polycythemia phenotypes are common in patients with advanced heart failure and present in 10% to 20% of the ambulatory heart failure population and may have worse clinical and functional status and an increased risk of prior and future thromboembolic events. Low volume phlebotomy is a safe and well-tolerated procedure which may benefit patient clinical outcomes. Further studies are needed to assess BVA’s ability to monitor these patients.

## Funding Support and Author Disclosures

Dr Silver is a Medical Advisor for Daxor. All other authors have reported that they have no relationships relevant to the contents of this paper to disclose.
